# A Vision-Based Odometer for Localization of Omnidirectional Indoor Robots

**DOI:** 10.3390/s20030875

**Published:** 2020-02-06

**Authors:** Cosimo Patruno, Roberto Colella, Massimiliano Nitti, Vito Renò, Nicola Mosca, Ettore Stella

**Affiliations:** Institute of Intelligent Industrial Technologies and Systems for Advanced Manufacturing, Italian National Research Council, STIIMA-CNR, G. Amendola 122 D/O, 70126 Bari, Italy; roberto.colella@stiima.cnr.it (R.C.); massimiliano.nitti@stiima.cnr.it (M.N.); vito.reno@stiima.cnr.it (V.R.); nicola.mosca@stiima.cnr.it (N.M.); ettore.stella@stiima.cnr.it (E.S.)

**Keywords:** visual odometry, vision-based odometer, affine pose estimation, computer vision, SURF, monocular camera, feature-based approach

## Abstract

In this paper we tackle the problem of indoor robot localization by using a vision-based approach. Specifically, we propose a visual odometer able to give back the relative pose of an omnidirectional automatic guided vehicle (AGV) that moves inside an indoor industrial environment. A monocular downward-looking camera having the optical axis nearly perpendicular to the ground floor, is used for collecting floor images. After a preliminary analysis of images aimed at detecting robust point features (keypoints) takes place, specific descriptors associated to the keypoints enable to match the detected points to their consecutive frames. A robust correspondence feature filter based on statistical and geometrical information is devised for rejecting those incorrect matchings, thus delivering better pose estimations. A camera pose compensation is further introduced for ensuring better positioning accuracy. The effectiveness of proposed methodology has been proven through several experiments, in laboratory as well as in an industrial setting. Both quantitative and qualitative evaluations have been made. Outcomes have shown that the method provides a final positioning percentage error of 0.21% on an average distance of 17.2 m. A longer run in an industrial context has provided comparable results (a percentage error of 0.94% after about 80 m). The average relative positioning error is about 3%, which is still in good agreement with current state of the art.

## 1. Introduction

Accurate localization and robust navigation are essential for most of mobile robot applications. Over the last decades, strong attention has been given to develop novel methodologies and algorithms for achieving autonomous navigation. An accurate pose estimation increases the capabilities of both robots/vehicles and autonomous systems in navigating properly inside the environment thus reducing the failure rate during missions. The start of the fourth industrial revolution, well known as Industry 4.0, is establishing new paradigms and concepts regarding to the robot navigation and localization, especially in logistics and transport [1]. The robots will not be any more constrained into limited working areas, but they will move and operate in different zones of factories and warehouses. In this regard, robust and reliable algorithms for localization purposes have to be devised.

**Figure 1:**
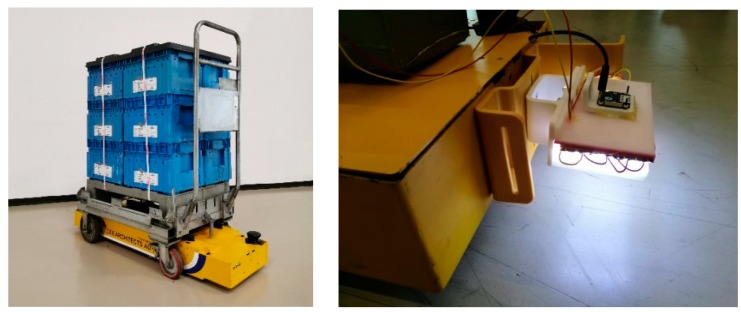
On the left, an autonomous omnidirectional vehicle being experimented in an industrial setting for moving carts between different zones of a factory; on the right, a close view to the vision-based system used to aid in robot localization.

In this paper, we propose a vision-based approach for estimating the relative pose of an omnidirectional automatic guided vehicle (AGV) used for transporting housing carts and finished goods carts around a large industrial building, starting from a known position and reaching some predefined areas. The objective is to introduce new technologies in support of better automation and productivity, consistently with Industry 4.0 philosophy, but without abruptly disrupting neither the current state of the workplace nor the workspace. This provides constraints in the design of navigation strategies (since it must manage to move in a shared environment with factory workers as well other “non-intelligent” vehicles) and, most important for this work, localization methodologies, since it limits the possibility to instrument the workspace. This leaves the localization duties to properly instrumented vehicles using proprioceptive sensors. This work targets the need of an AGV (see Figure 1) that has been provided to address the navigation problem in the industrial environment and that it was already proven being adequate in this context for performing maneuvers on the spot, when needed. A way to achieve this, and the one pursued in this work, is to exploit the texture information of the industrial environment, and in particular the factory floor, to perform localization tasks. The proposed vision-based odometer has been furtherly validated by considering different floor typologies in order to better investigate its accuracy performance.

The paper is organized as follows: Section 2 discusses the related works. Then, the explanation on how the proposed vision-based method works is presented in Section 3 whereas, the description of experiments and their outcomes are reported in Section 4. Final conclusion and future activities are in Section 5.

## 2. Related Works

Various techniques and sensors [2,3,4] as inertial navigation system (INS), global positioning system (GPS), global navigation satellite system (GNSS), monocular and stereo cameras, active beacons, RFID-based devices, odometers and so on, are employed to derive the robot pose information. The techniques for localization can be discerned in two main categories: relative and absolute localization. The absolute (or global) localization methods estimate the robot pose with respect to a global reference system or artificial or natural landmarks situated into the environment. On the contrary, the relative (or local) localization methods need the knowledge of initial location of robot for estimating its relative displacements over time. These techniques are well known as dead reckoning ones. The typology and topology of scenarios within the mobile robots operate and the required accuracies in terms of pose estimation, drive the choice of sensors and methods to use for attaining the autonomous robot navigation and localization. When the environment cannot be structured with additional sensors in order to preserve its integrity, the employable methods decrease even more.

The fast spread of computer vision (CV) has undoubtedly favored the development of several methods and relevant technologies for the localization and navigation of autonomous vehicles and robots in both indoor and outdoor environments. In this regard, visual odometry (VO) is currently one of the most effective and reliable technique adopted for relative localization [5,6,7]. The VO enables to incrementally estimate the pose of the robot by evaluating the appearance changes that the motion creates in the images captured by the passive cameras fastened on it. Differently to common wheel odometry (WO), the VO is not affected by non-systematic errors as wheel slippages mainly due to abrupt accelerations or uneven grounds. Consequently, the relative positioning errors of VO are much lower than WO errors. Nevertheless, the VO is a dead reckoning technique and possible errors introduced during the motion estimation by processing two consecutive frames involve drifts of the estimated trajectory from the actual path over time [8]. These errors accumulating over time can be periodically vanished by resetting the visual sensors or corrected by means of supplementary approaches.

Over the last years, newly methods and algorithms have been proposed for robot localization [9,10,11]. Some works explore the use of optical mouse sensors for developing positioning systems. In [12], a low-cost customized optical sensor is proposed where, an adaptation mechanism for enhancing the robustness to surface height variations and a novel calibration paradigm with fiducial landmarks used for mitigating the cumulative errors have been developed. Their prototype uses three optical mouse sensor units located at different heights along a slideway. The fusion of data from these sensor units helps to reduce the height variation errors. In [13], three optical mouse sensors placed under the robot according to a triangular-geometry configuration, have been combined with an encoder sensor of driving wheels in a Kalman filter-based framework for providing the positioning information and limiting both systematic and non-systematic errors.

The use of mouse sensors offers valid solutions and alternatives to wheel odometers for applications requiring accurate relative positioning measurements. Nevertheless, they suffer from some disadvantages. Firstly, they provide only displacement measurements along the ground surfaces with high frequency and accuracy neglecting mere rotation estimations. The integration of two or more mouse sensors is mandatory for deriving the rotation information. Secondly, their working distance is commonly restricted to few millimeters, thus limiting their applicability in common transportation systems. In fact, their functioning principle foresees that a very tiny portion of target surface is observed in order to relate its micro-structural information over multiple frames. 

When these micro-structural elements cannot be analyzed because of the surface properties or the particular application, a large-scale analysis of the scene is required.

Three main approaches can be used for estimating the pose of a mobile vehicle or robot through the VO: feature-based approach, appearance-based approach and hybrid-based approach [14,15,16]. The feature-based one takes advantage of the saliency and the repeatability of some keypoints to be tracked over the frames. The appearance-based (or global) one uses all the intensity pixel information of two consecutive images but are less accurate than the first category and computationally more demanding. The hybrid-based approach combines the methods of the other two categories.

Many algorithms have been proposed by the scientific community which use the feature-based approach for robot localization. In [17], a system that estimates the motion of monocular and/or stereo cameras is discussed. The Harris corner detector enables to locate the textureness maxima in the image and the feature points are then matched through multiple images using geometric hypothesize-and-test architecture for obtaining robust estimates of camera motion. A fast Hessian-based feature detector/descriptor (CVL) has been developed to refine the image localization of features in [18,19]. The motion is then estimated by using a robust bundle adjustment of matched feature points, which are independently extracted from two pairs of stereo images. The authors of [20] have used the SIFT (Scale Invariant Feature Transform) detector [21] and a data post-processing strategy for enhancing the motion estimates of a vehicle in complex urban environments.

The most recent developments include the adoption of modern learning techniques to overcome typical disadvantages of traditional computer vision methods (the need for careful parameters setting, high sensitivity to environment and illumination changes, image blur and other factors). In [22] authors propose a visual odometry (VO) system based on unsupervised deep neural networks trained over stereo images and tested with monocular images, that is able to estimate the 6-DoF pose of a monocular camera. Moreover, [23] involves the development of a convolutional neural network model for computing the relative motion between two frames in which both the training and test phases are carried out on a public dataset.

Generally, the VO techniques enable to estimate the robot or vehicle pose in terms of six degrees of freedom (6-DoF). However, when the cameras are oriented towards to the ground plane, the problem of pose estimation could be simplified to a planar rotation and translation of three degrees of freedom (3-DoF), when ground robots are considered. As an example, in [24] a single downward-looking camera has been fastened onto a ground vehicle for VO computation. The authors exploit the Ackerman-like steering model and the optical flow information for simplifying the motion estimation, which can be decomposed in a pure forward/backward translation and a pure rotation around the center of rear wheel axle. A similar configuration has been adopted in [25], where a rotated template matching approach is used for motion computation. A selected patch of variable size in the first image is rotated according to predefined angles and then compared with the second image. A multi-template correlation matching is presented in [26] where different quality responses as median, absolute deviations, entropy, cross-correlation, variance, are evaluated for selecting the best template candidate. The authors of [27] have proposed the fast Fourier transform (FFT)-based image registration method for determining accurate translational information of ground robot. The phase correlation of two input images is analyzed in order to find the impulse corresponding to the location of maximum translation. The rotation estimation is performed indirectly by converting the X-direction image translations in heading angles. In [28,29,30] an embedded vision system based on laser profilometry is used for obtaining the relative pose of an AGV that can move into a structured environment. The authors correlate the laser signatures extracted by processing consecutive images for estimating the relative displacements of robot along the movement direction.

The analysis of literature reveals that an extensive use of vision sensors has been done for VO applications. In this regard, many solutions based on mouse sensors have been proposed. Nevertheless, their applicability is limited to the application typology and more sensors need to be installed for obtaining the rotation estimates as well, thus requiring more available space to be mounted on the robots/AGVs.

In this work, we focus our attention on classical approaches that do not make use of machine learning techniques, in order to understand what it is possible to achieve with a monocular vision system while controlling all the other parameters (viewpoint perspective, illumination and so on). For the same reason, no other auxiliary sensors such as inertial mems units, are investigated in this paper. Moreover, other positioning-oriented sensors are already included in the omnidirectional AGV and the integration of our odometer with the rest of the system is scheduled at a later date.

A solution relying on typical cameras assisted with the suitable optical system enables to better manage different situations but still adopting the same processing strategies. A feature-based approach is proposed here for extracting the keypoints of interest, which enables to achieve better localization accuracy rather than the ones based on template matching or global analysis already presented in literature. A robust mechanism for rejecting the incorrect feature correspondences is presented together with a calibration procedure for compensating camera plane rotations with respect to the ground surface. This compensation enables to reduce the complexity of pose estimation problem thus implying more accurate displacement measurements computed from consecutive images.

## 3. Methodology

**Figure 2:**
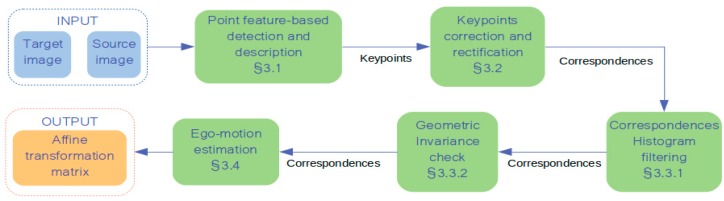
Processing pipeline employed in the methodology, starting from pair of successive images and providing the affine transformation matrix of the detected motion.

This section describes in details the whole processing pipeline used for developing our VO system. The feature-based approach is adopted for extracting the keypoints of interest from the flow of images. These keypoints are related to each other over consecutive frames by means of strong and robust descriptors. However, the matching phase might frequently provide incorrect associations because of the low-textured environment wherein the autonomous AGV works. Our robust correspondence feature filter enables to reject such incorrect correspondences thus enhancing the final relative pose estimation. Other processing steps are necessary as well. Figure 2 shows the full processing pipeline.

The first subsection will describe briefly the point features detector. Since lens distortions and other lens and camera parameters can affect the acquisition and ultimately the processing, camera calibration guided correction on detected keypoints is applied too and is discussed next. Correspondences rejector techniques are then applied, in the pursue of minimizing the negative effects that unreliable features can have on localization. The last part will be focused on the ego-motion estimation of the robot.

### 3.1. Point Feature-Based Detector and Descriptor

Over the years, many researchers have extensively studied and proposed methods for efficiently detecting robust features by analyzing images. Currently, the most effective keypoint detectors are the Scale Invariant Feature Transform (SIFT) [21], the Speeded-Up Robust Features (SURF) [31], the Binary Robust Invariant Scalable Keypoints (BRISK) [32] and the Features from Accelerated Segment Test (FAST) [33]. The SIFT, SURF and BRISK methods propose also descriptors for extracting the characteristics of the surrounding region related to the detected keypoints. Another highly discriminative binary descriptor, such as the Binary Robust Independent Elementary Features (BRIEF) [34], allows to efficiently reduce the required processing time during the matching stage.

Among the proposed methods, our VO algorithm uses the SURF approach for locally detecting distinctive, robust and invariant points given an input image. This feature is partly inspired to the SIFT method but it performs much better than its counterpart in terms of rotation, blur, warping, RGB noise and time consuming [35]. Since the SIFT method is time consuming, the authors of SURF have proposed a speeded-up version still ensuring very high performance. Since the convolution operation is computationally expensive, the use of both the integral image and the box filters enables to speed-up the computation of scale-space keypoint locations. The Laplacian of Gaussian (LoG) along the *x*-direction, *y*-direction and the *xy*-direction, is discretized by using kernels of different sizes. Consequently, the scale space domain is evaluated by up-scaling the box filters rather than down-sampling iteratively the image. In SURF, the lowest level, or conversely the highest spatial resolution, of the scale space is obtained by using 9 × 9 box filters, which are approximations for Gaussian second order derivatives having standard deviation equal to 1.2. This approximation enables to convolve the integral image with the box filters in an easily way and in parallel for different scales. The keypoint detection relies on the computation of the determinant related to the approximated Hessian matrix computed by considering the convolutional outputs of considered box filters. Among all points of image, only the ones providing maximal determinant values are considered. Specifically, these maxima are interpolated in both image and scale space for detecting the precise sub-pixel location of keypoints. The keypoint orientation is assigned by using the Wavelet responses along the horizontal and vertical direction by analyzing its surrounding region having size 6 *s* (where *s* stands for the found scale value). The Wavelet analysis is performed by employing *2*-dimensional Haar filters. The dominant orientation can be computed by calculating the sum of all the responses within a sliding angular window. The SURF descriptor takes advantage once more of the Wavelet analysis by considering a keypoint neighborhood of size 20 × 20 s, opportunely aligned according to the found dominant orientation. This region is divided in 4 × 4 patches. Each patch returns back four values that represent the Wavelet responses. The final SURF descriptor has a size of 64 elements. A 128-dimension version of descriptor is also available, where the sign of responses is considered as well. It is worth noting that in the detection of relevant keypoints, this work takes special care while considering possible features near the image borders. While the SURF keypoint is detected by analyzing a region of size 15 *s*, its corresponding descriptor is computed by considering a wider area having size of 20 *s* [36]. Therefore, those keypoints located close to the image borders can produce descriptors whose support region can cross the borders. In this situation, different techniques (e.g. image extension using reflection or closest pixel values, zero padding, etc.) can be used for obtaining the missing data outside the borders, although the computed descriptors could be not more adequate for matching purposes. Consequently, the keypoints near to the borders are opportunely discarded.

### 3.2. Keypoints Correction and Rectification

Acquisition is naturally affected by unwanted distortions introduced by the imaging setup that can change the perceived distance between feature points. It is therefore important to address these issues by exploiting a camera calibration with data collected using a calibration pattern. It is worth noting here that, for reasons related to practical implementation and speed, only the detected keypoints on the images pair are corrected in this work, hence this processing step happens here and not at the beginning of the pipeline, as usually is the case. The primary objective of the rectification in this work is to remove unwanted distortions. Moreover, a clear distinction between lens, intrinsic and extrinsic parameters is done only when the distinction has been used in some ways (better performance/ accuracy/simplifications/etc.).

Other characteristics related to the context in which the system is designed to be employed can be considered as well, since limiting indoor navigation to a structured environment enables to impose additional constraints, thus resulting in better relative pose measurements. In particular, differently from other VO approaches, the proposed one allows to provide the 3-DoF robot pose by processing consecutive images instead of the full 6-DoF pose. Since the monocular camera is oriented towards the ground surface with its optical axis nearly perpendicular to this one and the AGV does not own suspension systems, it is possible to assume that the height of the vision system will be always fixed, thus never changing its value during motion. Consequently, the scale factor does not require to be estimated because of the prior knowledge of distance between the acquiring vision system and the target planar surface, thus simplifying the pose computation. In the event that an ideal case is considered, i.e. when the image plane and the ground plane are perfectly coplanar, the height value is the same for all the pixels of the image. Nevertheless, in real cases this assumption is no longer satisfied since ensuring the mechanical parallelism of these planes is very complex. Minimal tilt differences of planes very likely result in increased measurement errors in the relative pose estimation without a proper alignment calibration step. These errors progressively accumulate over time thus leading to worse results.

**Figure 3:**
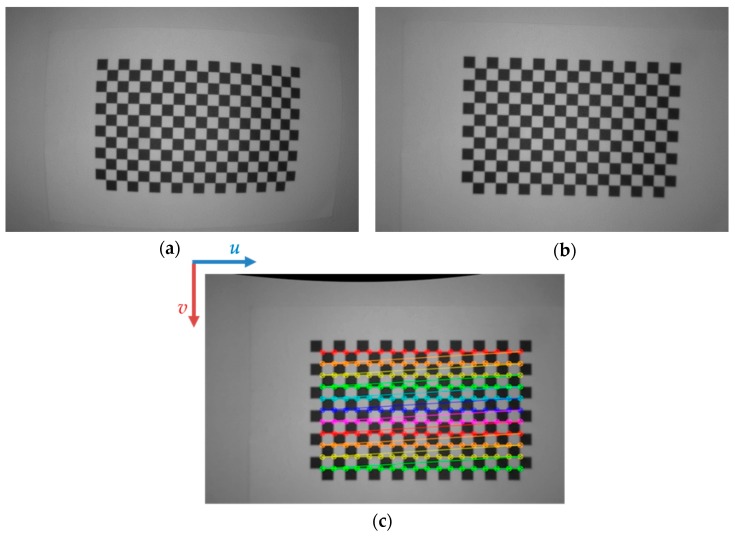
Calibration pattern placed on the ground floor before (**a**) and after (**b**) the rectification step. The camera pose compensation of rectified image (**b**) is evident in (**c**) as it leads the chessboard corners to be well aligned with the horizontal *u-* and vertical *v-* directions of the image under investigation.

In order to reduce these unwanted effects, two calibration steps are performed: the first one is aimed at correcting the distortion effects due to the curvature of adopted lenses [37] and the second one performs the precise alignment between the image plane and the ground plane, which is assumed to be planar without strong deflections. By using the standard calibration procedure [38] which employs a chessboard-based pattern, it is possible to find the unknown parameters as the intrinsic camera matrix *K* and the distortion coefficients *D* (please refer to Equation (1)). By knowing the location of chessboard corners and by detecting them over different images captured by varying the pattern pose, it is possible to solve a non-linear equation system for estimating these parameters. Afterwards, the 2D image points can be rectified. As an example, Figure 3a reports the calibration pattern lying on a planar surface, whose sample image has been opportunely rectified (please refer to Figure 3b). As one can notice, the distortion effects are efficiently mitigated and the edges of the pattern that initially appear as curves, become straight lines as expected. This also addresses (at least partially) perspective distortions since the calibration pattern is rectified once the calibration correction is applied. Indeed, the pose rectification can be performed by exploiting the extrinsic information. Specifically, the chessboard has to be placed on the planar floor in order to estimate what is the camera pose (already fastened on the mobile robot) with respect to the calibration pattern. As a consequence, it is possible to understand how the camera is oriented with respect to the ground plane. A *4x4* extrinsic matrix *M* (see Equation (1)) can be thus easily estimated, whose rotation information is used for opportunely re-mapping the 2D image points. In this way, slight angular variations of camera pose can be better compensated. This provides better positioning accuracy.

(1) $$K=\left[\begin{array}{ccc} f_{x} & 0 & c_{x}\\ 0 & f_{y} & c_{y}\\ 0 & 0 & 1 \end{array}\right];\;D=\left[\begin{array}{cc} \begin{array}{cc} \begin{array}{cc} \begin{array}{cc} d_{1} & d_{2} \end{array} & d_{3} \end{array} & d_{4} \end{array} & d_{5} \end{array}\right];\;M=\left[\begin{array}{cccc} r_{11} & r_{12} & r_{13} & t_{x}\\ r_{21} & r_{22} & r_{23} & t_{y}\\ r_{31} & r_{32} & r_{33} & t_{z}\\ 0 & 0 & 0 & 1 \end{array}\right]$$

As effect of this pose compensation by means of the estimated rotation matrix, the sample image reported in Figure 3b is remapped as in Figure 3c. This leads the chessboard corners to be perfectly aligned with the horizontal *u-* and vertical *v-* directions of the image under analysis.

At this stage, the keypoint locations are corrected from the distortion effects and their pose rectification is opportunely applied.

### 3.3. Correspondences Feature Filter

Despite the SURF approach is being very effective for the most of CV-based and VO-based applications, sometimes the typology of environment characteristics might affect negatively the robust and accurate correlation of the keypoints over images by means of the related descriptors. As an example, the occurrence of repetitive patterns in the scene of analysis or/and low-textured surfaces might produce quite similar descriptors, thus involving incorrect and unwanted matchings. Therefore, a robust method way for filtering out those wrong point correspondences has been developed. Filtering consists of two steps: histogram filtering and geometric invariance check.

#### 3.3.1. Correspondences Histogram Filtering

**Figure 4:**
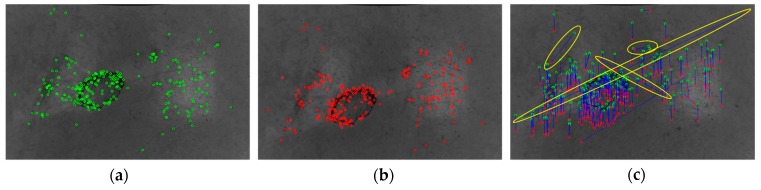
Detected keypoints related to the (**a**) target image and (**b**) source image. (**c**) Corresponding keypoints related to two consecutive frames acquired during the motion. Some incorrect matches to be removed are highlighted by yellow ellipses.

Figure 4 reports the detected keypoints obtained by processing two consecutive images acquired during the motion of the mobile robot or AGV. The green circles represent the keypoint locations on the first image (or target image in Figure 4a) whereas the red circles stand for the keypoint locations related to the second image (or source image in Figure 4b). Keypoints correspondences are shown with a blue connection line in Figure 4c, where keypoint descriptors are taken into account. However, as highlighted by the yellow ellipsoids, some of the reported correspondences are incorrect.

In order, to filter out those incorrect 2D point associations, a histogram analysis is performed. In this work, rotations and translations are considered separately, one after the other.

**Figure 5:**
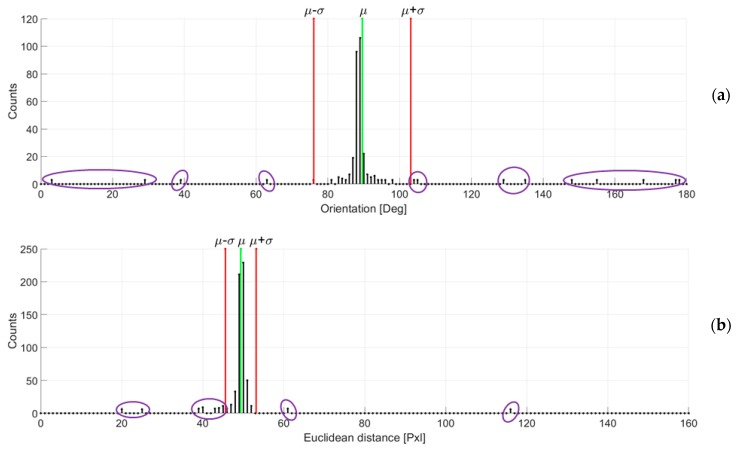
Orientation histogram (**a**) and Euclidean distance histogram (**b**) computed by considering the one-to-one point correspondences for the case under investigation. The extraction of statistical information related to the two histograms enable to efficiently filter out the outlier points. The variables μ and σ represent the average value and the standard deviation referring to the considered distribution.

Firstly, all the orientations of joining straight lines are cumulated into an array having 180 bins equally spaced. In this way, a histogram of orientations can be formed, with each bin covering one degree. The peaks of this histogram indicate the dominant orientations. Once the histogram is filled, a statistical analysis enables the preliminary removal of the outliers. With reference to Figure 5a, the histogram related to the keypoints under investigation is reported, where some outliers, out of the statistics, are highlighted in purple ellipses.

Secondly, a similar statistical analysis is performed for the remaining inlier correspondences from previous step by considering their Euclidean distance. In this way, too long or too short segments (or joining lines) are efficiently removed because they do not meet the expected distance criterion. The Euclidean distances are cumulated into a histogram by considering 160 bins. The number of bins has been set empirically, taking into consideration the image size and the need for a feature to be visible for at least two consecutive frames. Figure 5b shows the Euclidean distance histogram, whose statistical analysis aids to filter out the incorrect matches (once more in purple). A gaussian distribution is considered for analyzing both histograms.

The choice of a gaussian distribution is driven by the fact that, given the floor facing sensor, expected motion should be coherent across the whole surface, although affected by noise that might introduce measurement errors. A mixture distribution is therefore unfeasible for modeling the problem. In the absence of specific data that might suggest otherwise, a gaussian distribution is a good candidate to analyze the data.

**Figure 6:**
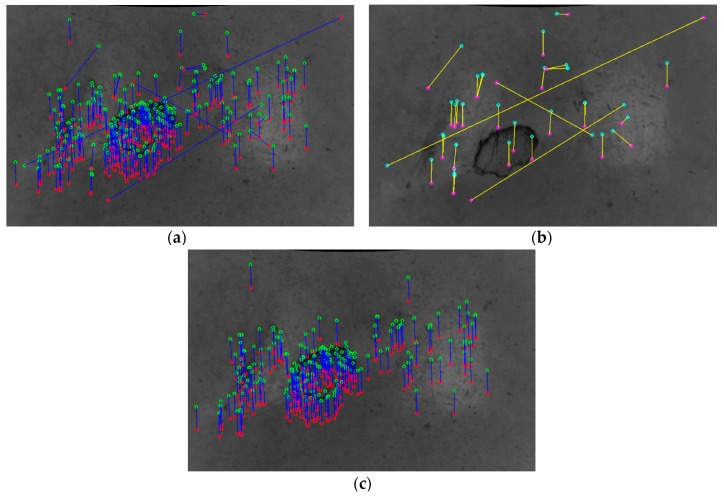
Corresponding keypoints (**a**) before and (**c**) after the application of statistical method. (**b**) Corresponding matchings being filtered out from the following processing.

At this stage, all the correspondences that do not meet the statistical requirements of both distance and orientation, i.e. the ones having values out of the range (μ−σ,μ+σ) where μ and σ stand respectively for the average value and the standard deviation related to the considered distribution, are removed from the following processing. Figure 6a shows the correspondences that undergo the statistical analysis and Figure 6b reports the incorrect matchings that are removed. In Figure 6c the remaining correspondences are shown, that proceed in the next step of the analysis.

#### 3.3.2. Geometric Invariance Check

**Figure 7:**
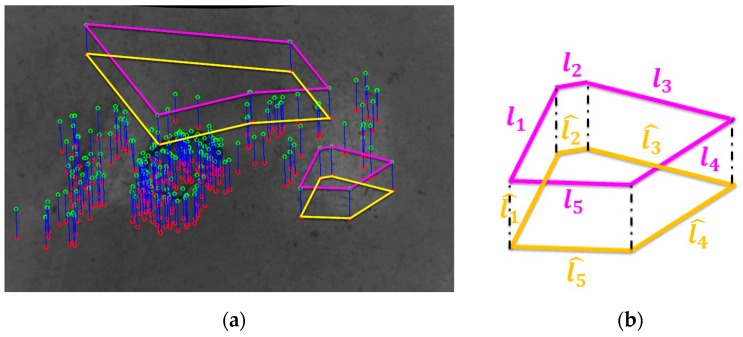
(**a**) Some polylines having five sides are built by using the target and the source keypoints. (**b**) Enlargement of polylines in relation with each other, whose edge lengths have to be evaluated for establishing the goodness and robustness of keypoints in terms of image locations.

Despite the keypoints appearing correct after the previous filtering steps (see Figure 7a), some unreliable features can still be present. Specifically, slight lit condition variations, small perspective deformations or distortions of images, or even numerical approximations, might affect negatively the accurate extraction of point locations. Consequently, we developed a further filtering method for detecting the best feature correspondences among the extracted ones. A geometrical analysis allows to robustly reject the keypoints that do not meet specific criteria. This correspondence rejection algorithm takes advantage of the pose invariant geometric constraints between the target keypoints and the source keypoints related to solid or not-deformable surfaces, as is the case for the floor of indoor environments. In this way, virtual closed line loops (CLL) can be formed, where the randomly selected features define a polyline. The idea is that the distance between successive pairs of points of each set must be preserved, if the correspondences are correct. A further check is considered for the distance between the first point and the last point of each set, thus forming a closed loop. The cardinality *C* of a single line loop in terms of its side number is dynamically tuned according to the number of input correspondences. Once the cardinality is computed, the method randomly selects different groups of keypoints, where each group contains *C* keypoints uniquely chosen from the source set. At this stage, the CLL related to a group of keypoints can be easily built, where the keypoints represent its vertices. The corresponding CLL built by using the related keypoints of the target set as vertices, has to present similar shapes and the side lengths must be approximatively equal. By opportunely thresholding the edge length differences it is possible to discard from the next processing all the keypoints weakly located. Figure 7a reports some examples of extracted polylines having five sides. The pink polylines are built from the source keypoints and the corresponding orange ones are formed by considering the related target keypoints.

With reference to Figure 7b, it is possible to define Equation (2) for evaluating the location robustness and the reliability of feature points.

(2) $$\frac{\min\left(l_{i},{\hat{l}}_{i}\right)}{\max\left(l_{i},{\hat{l}}_{i}\right)}\geq t\overset{}{\forall i};i=1,\ldots ,C$$

**Figure 8:**
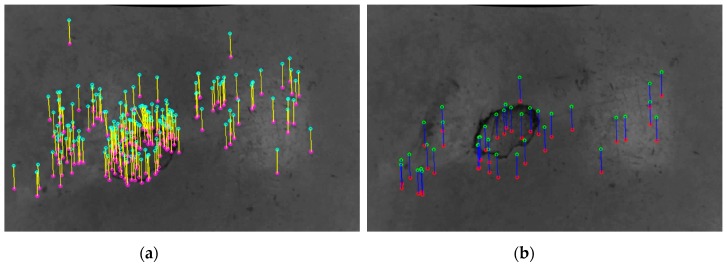
(**a**) Rejected and (**b**) good correspondences by applying the geometric invariance check approach. The parameter *t* is set to 0.85 and a cardinality *C* equal to three is considered.

In case all the edge lengths of two corresponding polylines is almost similar because of the pose invariant assumption, the related keypoints are labeled as good points. Conversely, all the keypoints considered in the closed line loop are discarded from the final point sets if just one of the considered distances fails the test according to Equation (2). The ratio between two corresponding sides is always in the range [0,1]. Figure 8 reports the correspondences labeled as outliers by means of the proposed geometric invariance checks and the remaining robust correspondences.

The main steps for checking the geometric invariance between the keypoints are summarized in Algorithm 1.
**Algorithm 1** Geometric invariance check**Input**: *K_S_*, *K_T_*, *t*. **Output**: *K_S__out*, *K_T__out*.1.*K_S__out* = [], *K_T__out* = []2.*n* = Size(*K_S_*);3.*C* = ComputeCardinality(*n*);4.[*G_S_*, *G_T_*] = RandomSelection(*K_S_*, *K_T_*, *C*);5.**For each** item *i* of *G_S_* **do**6. *CLL_S_* = ComputeCLL(*G_S_*[*i*]); *CLL_T_* = ComputeCLL(*G_T_*[*i*]);7. *Count* = 0;8. **For each** item *j* of *CLL_S_* **do**9.  **If** $\left(\frac{\min\left(CLL_{S}\left[j\right].Length,\;CLL_{T}\left[j\right].Length\right)}{\max\left(CLL_{S}\left[j\right].Length,\;CLL_{T}\left[j\right].Length\right)}\geq t\right)$ **then**10.   *Count* = *Count* + 1;11.  **End If**12.  **If** (*Count* == C) **then**13.   Insert *G_S_*[*i*] in *K_S__out*; Insert *G_T_*[*i*] in *K_T__out*;14.  **End If**15. **End For**16.**End For**17.**Return***K_S__out*, *K_T__out*.

### 3.4. Ego-Motion Estimation

The last step computes the ego-motion of the AGV or mobile robot by considering the extracted and then filtered keypoints from previous stages. The 2D keypoints are converted in metric units by taking advantage of the intrinsic camera matrix *K* and the extrinsic matrix *M* containing the distance information $t_{z}$ between the image plane and the ground plane.

At this point, the two sets of 3D keypoints are used for estimating the relative motion information between two consecutive frames by solving a non-linear equation system (please, refer to Equation (3)). Since the *z*-coordinate is the same for all the projected keypoints due to the tilt camera compensation, the estimation can be furtherly constrained. In this way, the 3*x3* affine matrix that aligns the source keypoints to the target keypoints can be easily computed.

(3) $$\left[\begin{array}{c} x_{t}\\ y_{t}\\ 1 \end{array}\right]=\left[\begin{array}{ccc} \cos\theta & -\sin\theta & T_{x}\\ \sin\theta & \cos\theta & T_{y}\\ 0 & 0 & 1 \end{array}\right]\left[\begin{array}{c} x_{s}\\ y_{s}\\ 1 \end{array}\right]$$

The vectors ${\left[\begin{array}{ccc} x_{t} & y_{t} & 1 \end{array}\right]}^{T}$ and ${\left[\begin{array}{ccc} x_{s} & y_{s} & 1 \end{array}\right]}^{T}$ represent the homogeneous metric locations of target and source keypoints, respectively. The motion information are encoded in the affine matrix, where $T_{x}$ and $T_{y}$ stand for the planar translation of AGV along the *xy*-axes and the parameter θ is the rotation angle by considering the two input images.

Each pair of images returns an affine transformation matrix $A_{i}^{i-1}$ after the application of proposed processing steps. By gradually composing multiple affine matrices obtained when the robot moves in the environment by using Equation (4), it is possible to derive its final pose encoded into the matrix $A_{i}^{0}$ and thus reconstructing the motion trajectory.

(4) $$A_{i}^{0}=A_{1}^{0}\cdot A_{2}^{1}\cdot A_{3}^{2}\cdots A_{i-1}^{i-2}\cdot A_{i}^{i-1}$$

## 4. Results

The effectiveness and the accuracy of proposed method have been evaluated through several experiments by considering different floor typologies. Both qualitative and quantitative validations have been done. The following sections will describe briefly the experiments and present the related results.

### 4.1. Experimental Setup

**Figure 9:**
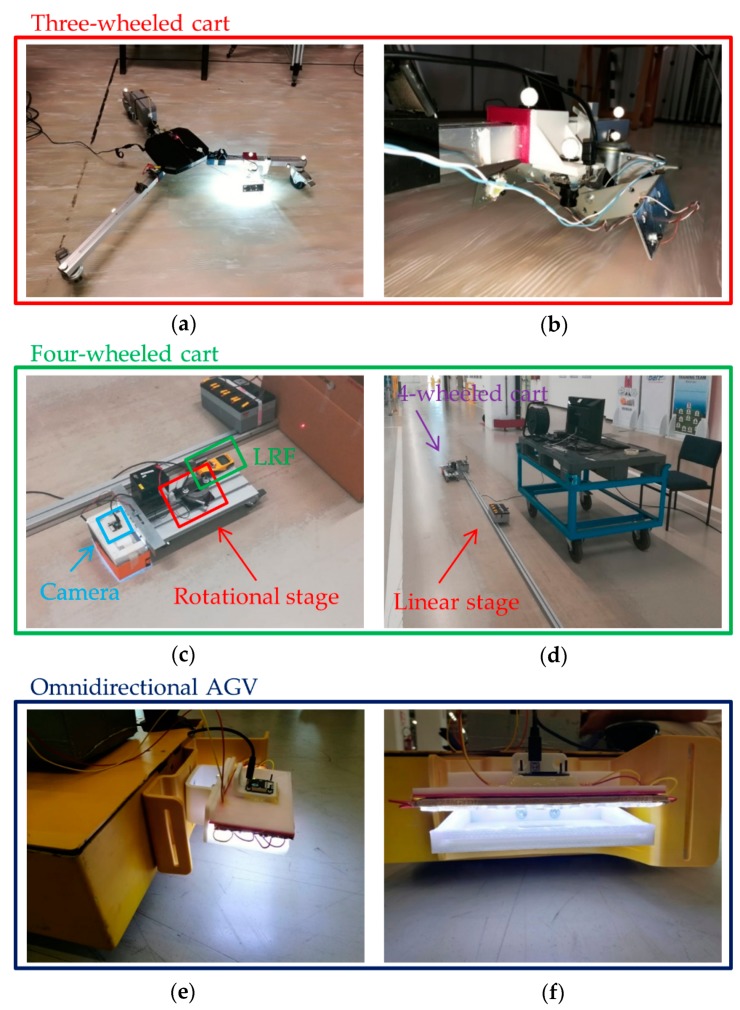
(**a**) Top view of the experimental three-axle cart and (**b**) side view of the lighting system together with the monocular camera and the spherical Vicon markers. (**c**) Four-wheeled cart, rotational stage and Laser Range Finder (LRF) for obtaining the ground truth and (**d**) global setup including the linear stage used with the four-wheeled cart. (**e**) side view of the vision-based odometer fastened onto the omnidirectional AGV and (**f**) front view of the lighting system together with the monocular camera.

The camera used during the experiments is the mvblueFOX-MLC200w [39] made by Matrix Vision having a resolution of 752 × 480 pixels with a pixel size of 6 μm. The used lens is the Lensagon BT3620 [40] by Lensagon having focal length of 3.6 mm. The monocular sensor has been fastened on two different moving carts, i.e. a three-wheeled cart and a four-wheeled cart and then on the target omnidirectional vehicle, as reported in Figure 9. All the processing algorithms are programmed in C++ with the support of OpenCV libraries (release 4.0 with contributes) [41] in order to ensure fast and efficient analysis of images. The onboard processing unit where the code runs is the Jetson AGX Xavier [42].

The four-wheeled cart, differently to the three-axle cart, enables to perform more controlled movements. In this way, it has been easier to get the ground truth of motion in those environments where an external tracking system (as the Vicon tracker) was not available. In some experiments the Vicon system has been adopted for obtaining very accurate measurements. This system includes eleven infrared cameras Vicon Bonita B10 [43] located around the area where the target can move. These cameras are able to detect specific spherical markers placed on the three-wheeled cart to be tracked, providing with high accuracy their 3D spatial information. A local reference frame can be established using a group of markers (see the white reflective markers in Figure 9a,b) to be associated to the camera which can be consequently tracked by the system over time. It generally operates up to a frequency of 400Hz achieving millimeter resolutions. When the Vicon tracker cannot be employed, linear and rotational stages together with one dot Laser Range Finder (LRF) [44] are used for obtaining the ground truth. Specifically, the LRF has an operating range from 0.1 m to 10 m and a precision of 1 mm (please refer to Figure 9c,d). The linear stage is mainly used for ensuring straight displacements of moving cart. Finally, in Figure 9e,f is reported the setup used for the omnidirection AGV (made available by courtesy of CodeArchitects Automation), which is the same of four-wheeled cart.

As the method takes advantage of the point feature-based approach, a good lighting system is mandatory for reducing the blurring effects and better highlighting the microstructures of the floor. Better light conditions involve more robust detection of keypoints, thus enhancing the accuracy of relative pose estimations.

In the experimental setup related to the three-wheeled cart we have used two matrix-shaped LED diodes [45] that transversally light up the ground plane (see Figure 9b for more details). However, when the floor is prevalently uniform and presents occasionally textured patterns, this solution is not adequate anymore. Consequently, a different solution has been considered for empathizing those microstructures as scratches or the less visible appearance features. Different flexible LED strips are adjacently fastened on a flat support. As our approach relies on the intensity analysis of pixels for extracting the keypoints, a uniform light distribution is required in order to avoid shadows leading to false feature detection. As a consequence, a satin plexiglass sheet is placed under the illumination system for uniformly spreading the light on the floor, as reported in Figure 9e,f.

**Figure 10:**
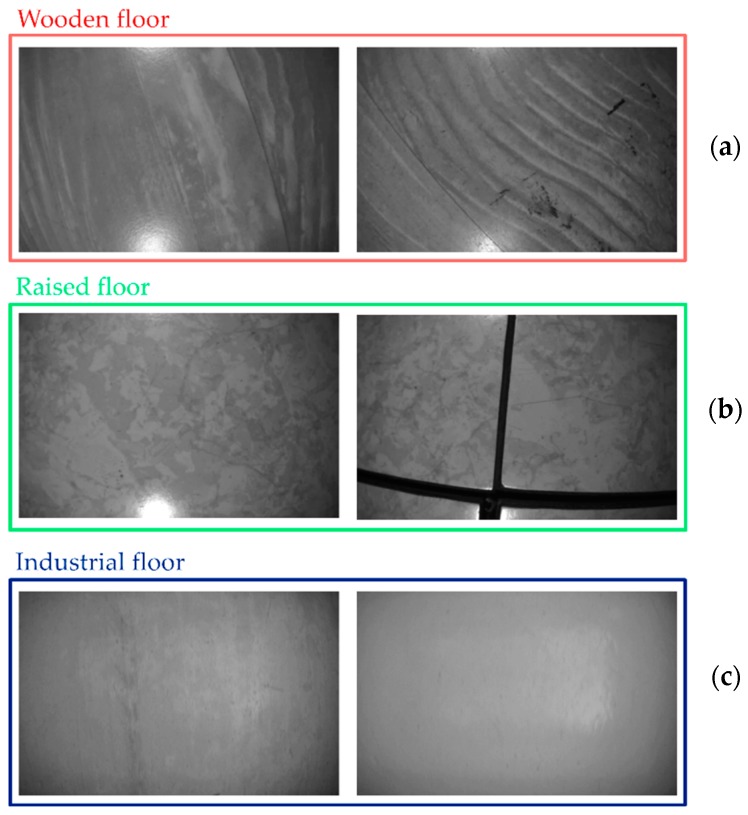
Some images captured by the camera related to the (**a**) wooden floor, (**b**) raised floor and (**c**) industrial floor. The industrial floor presents low (on the left) and very low (on the right) texture.

Finally, it is worth noting that the lighting system fastened on the three-wheeled cart produces two light spots on the floor, as observable in Figure 10a,b (please see at the top and the bottom of reported images). The accuracy of keypoints location can be affected negatively by the presence of these spots. Therefore, a suitable binary mask has been used for discarding all pixels related to these two areas during the keypoints detection phase.

### 4.2. Validation of Proposed Approach

The experiments are mainly grouped according to the typology of floors that the vision system frames. Specifically, three floor types are considered: wooden floor, raised floor and industrial floor. Each floor has different characteristics in terms of reflectivity, textureness, appearance and so on. In this way, the proposed approach can be evaluated by considering different challenging situations that occur when highly-reflective floors are framed.

In Figure 10, some images related to the considered floors are reported. As one can notice, the industrial floor seems to be the most challenging for the proposed vision approach as it is highly reflective and presents a low texture level in comparison with other two floors. The raised floor is averagely reflective but contains more patches and dark edges that outline adjacent floor panels. Finally, the wooden floor is low reflective but highly textured.

The remainder of this section will present the obtained outcomes. The Table 1 briefly summarizes the main details on how the sections are divided and which experiments will cover.

#### 4.2.1. Experiments with the Wooden Floor

Two tests have been run by moving the three-wheeled cart into our robotics lab having a wooden floor (see Figure 10a) where the motion tracking information is available as well.

**Figure 11:**
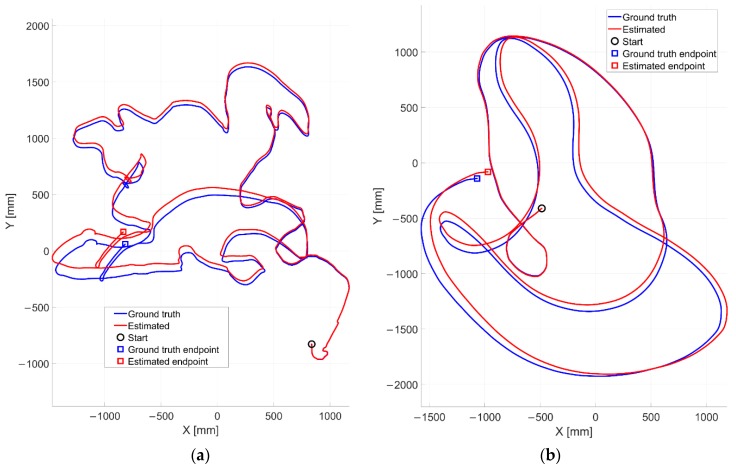
Estimated positions (in red) qualitatively compared with the ground truth ones (in blue) for (**a**) the experiment I and (**b**) the experiment II, both run at our robotics lab.

In Figure 11, the outcomes related to the experiments I and II are reported. The camera is moved randomly into the room by imposing combined translational and rotational displacements. As one can notice, in both the tests the estimated trajectories by means of our solution look qualitatively very similar to the reference ones, thus proving the effectiveness of proposed method.

**Figure 12:**
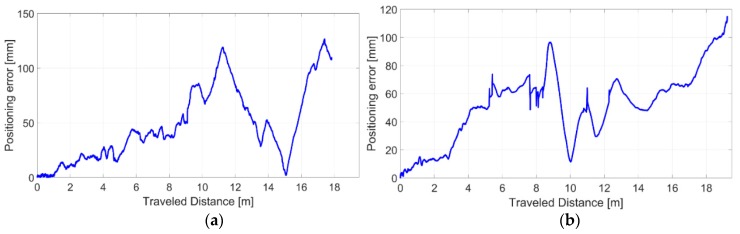
Drift positioning errors related to the first (**a**) and the second (**b**) experiment.

A quantitative analysis (refer to Table 2 in Section 4.3) shows average positioning RMSE values of 56.3 mm and 56.5 mm over a traveled distance by the moving cart of 17.83 m and 19.27 m for the experiments I and II, respectively. The three-wheeled cart has been moved at an average speed of 162.51 mm/s and 293.70 mm/s. It is worth highlighting that as all the dead reckoning navigation methods, our VO approach suffers from the inevitable drift errors on pose estimates. Consequently, the drift error is initially negligible (almost tending to zero) and it will gradually increase as the robot travels into the environment. In fact, the two trajectories (refer to Figure 11) which are initially well overlapped, are progressively diverging as effect of cumulated errors over time. This aspect is furtherly confirmed by observing to Figure 12, where the error signals tend to progressively increase, although of a small margin. As all dead reckoning techniques, this one requires periodically the pose restore as well.

The final positioning errors are found equal to 109.8 mm and 114.7 mm for the experiment I and experiment II, respectively. It can be noted that in some cases the positioning errors during the motion are higher than the final positioning error (e.g. in Figure 12a). This is due to how the error builds up over time. Since the error is not-systematic and can be positive or negative along the X- and Y- directions according to the motion type, the signal positioning error is not a strictly increasing or decreasing monotonic function.

**Figure 13:**
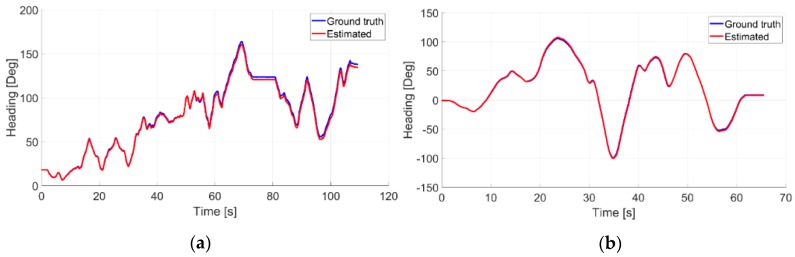
Angular estimations (in red) for the (**a**) experiment I and (**b**) the experiment II together with the respective ground truth ones (in blue).

A qualitative evaluation of presented approach is also provided by analyzing the heading estimates. With reference to the Figure 13, it is possible to observe how the estimated headings of vision system are very similar to the reference ones given by the Vicon system.

As observable, the drift angular error in the second experiment seems to be lower than the one related to the first experiment. This is probably due to the typology of imposed camera movements, which are more regular and smoother for the second test, as appreciable in Figure 11 as well. Further proves are given by observing the average heading RMSE values which are found equal to 2.3 degrees and 1.4 degrees, respectively.

#### 4.2.2. Experiments with the Raised Floor

**Figure 14:**
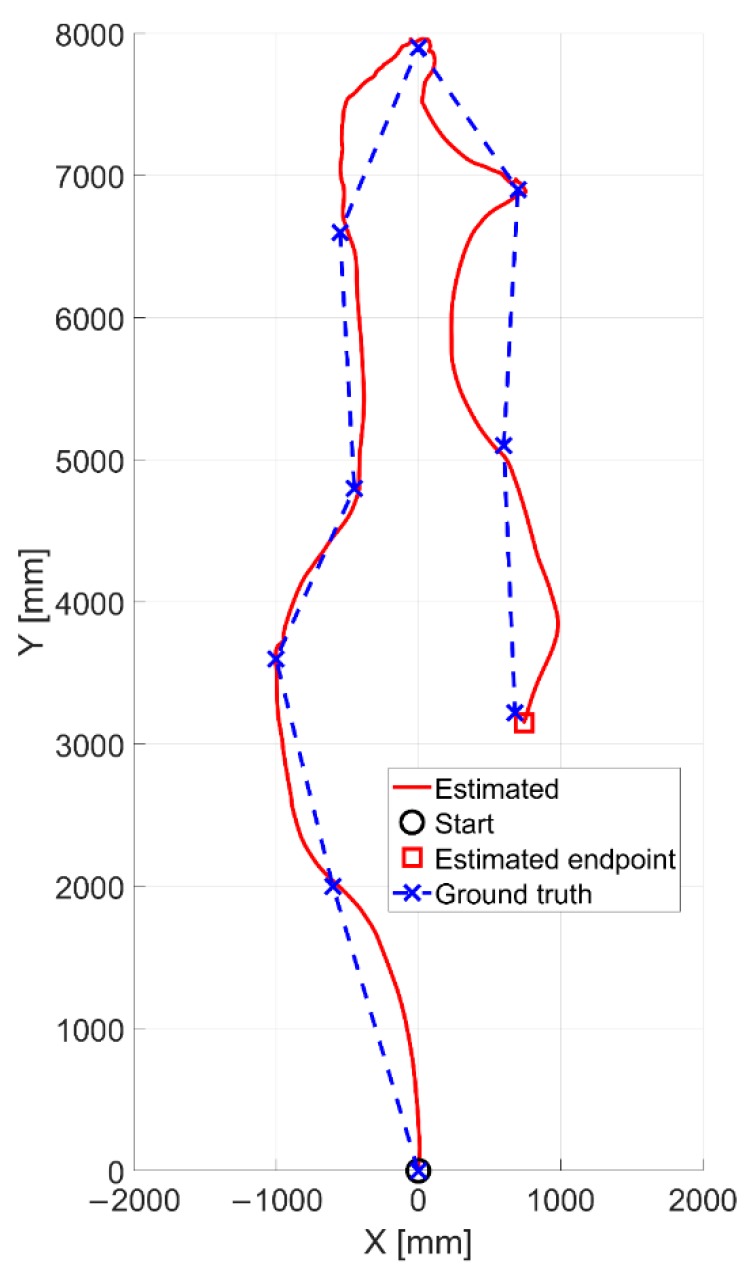
Estimated position of optical sensor (in red) and corresponding ground truth (blue markers).

The second part of validating experiments has been performed by moving the vision encoder along the corridor of our institute having different patterns and appearance floor, as shown in Figure 10b. In this case, we have placed specific markers on the floor at known locations for obtaining the ground truth. The optical sensor has been moved by following the path made by these markers. Nevertheless, the motion between two consecutive marks is unknown and thus pointing out the ground truth is not more challenging. Consequently, the ground truth is represented with dashed lines and crosses with reference to Figure 14, where the blue crosses stand for the true marker locations. The estimated positions are very close to the expected ones, thus proving once more the robustness of proposed algorithm in giving back accurate relative measurements. The average positioning RMSE value is equal to 42.8 mm over a traveled distance of 14.48 m. The average speed of the three-wheeled cart is 301.66 mm/s.

**Figure 15:**
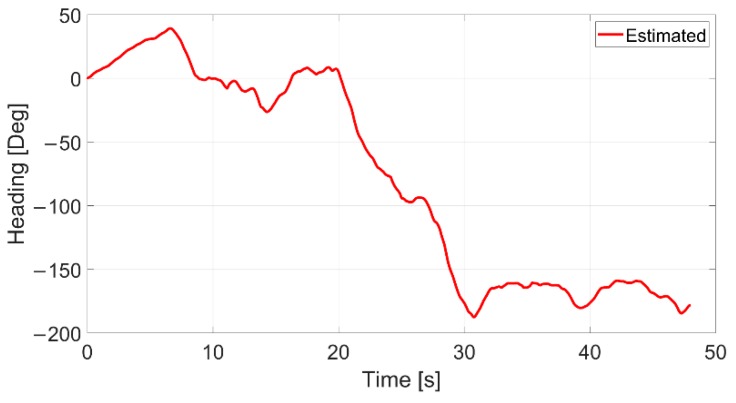
Estimated angle of optical sensor (in red).

For the sake of completeness, the heading estimates for this test are reported in Figure 15 as well. Also, in this case the angular signal looks very smooth without the presence of spurious spikes, that generally appear when erroneous pose estimations are computed.

#### 4.2.3. Experiments with the Industrial Floor

In this section, we report the experiments related to the third category of investigated floors, i.e. the industrial one (refer to Figure 10c for more details). Several tests have been performed at Bosch Diesel Systems SpA of Italy by using the four-wheeled cart on which the vision sensor has been fastened (refer to Figure 9c,d). By using the LRF together with rotational and translational stages, it has been possible to get the ground truth. For the sake of clearance, only the most relevant tests have been reported in this section.

**Figure 16:**
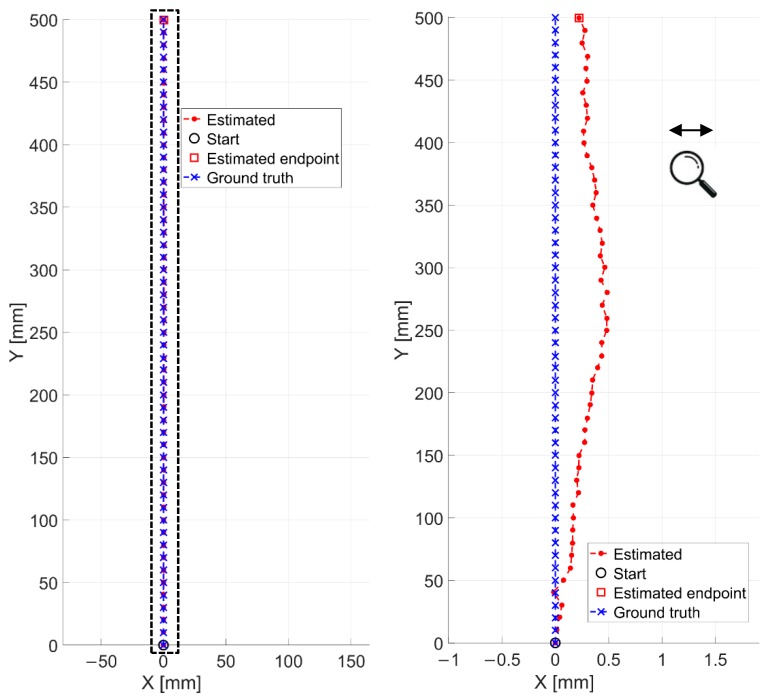
Estimated trajectory by our vision odometer (in red) and ground truth (in blue) related to the experiment IV. A zoomed in view (on the x-axis) is reported on the right for better appreciating differences among the two signals on the left (in dashed rectangle).

Controlled displacements and rotations have been performed. The experiment IV, as already reported in Table 1, has been run by considering the industrial floor having a low textureness and few features (see on the left of Figure 10c). The four-wheeled cart is moved by step of 10 mm by following the linear support reported in Figure 9c,d. The LRF is in charge of providing the ground truth in terms of distances from the starting point. An overall distance of 0.50 m has been covered. The results of this experiment are reported in Figure 16. Also in this case, the ground truth has been presented by means of blue crosses.

Qualitatively the estimated trajectory looks very comparable with the reference one. In this regard, an average RMSE value of 0.49 mm is found whereas, the final positioning error is about 0.35 mm. In order to better appreciate the differences between the two signals, a zoomed in view (on the x-axis) of the trajectory is reported on the right of Figure 16. One can notice that a maximum discrepancy of only 0.5 mm has been obtained, by proving once more the robustness and the accuracy of the method.

**Figure 17:**
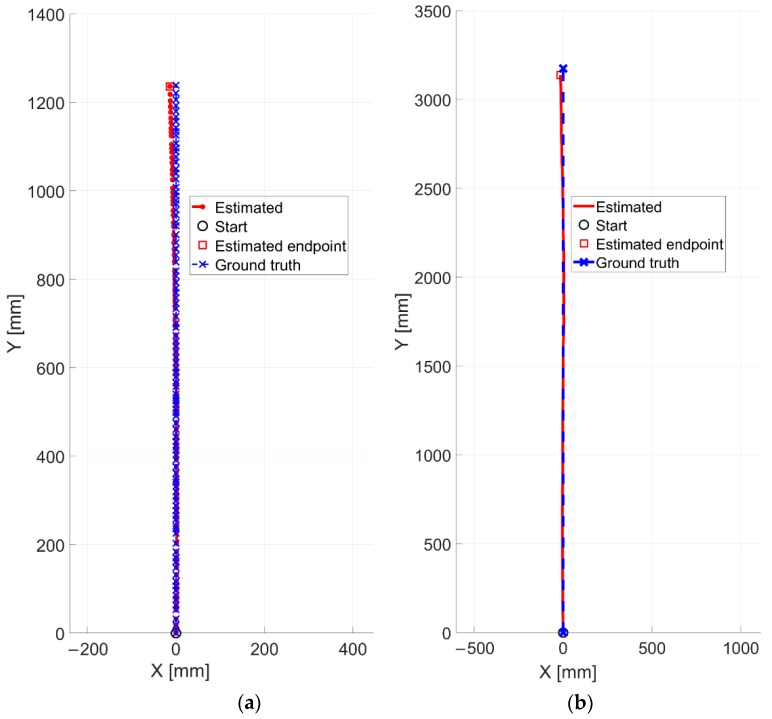
Estimated positions (in red) compared with the ground truth (in blue). The outcomes are related to the experiments (**a**) V and (**b**) VI.

The experiment V is similar to the previous one. However, in this case the cart is moved by variable steps by covering a distance of about 1.24 m. The industrial floor related to this test presents very low features (see on the right of Figure 10c) thus resulting more challenging for the vision-based odometer. The qualitative result is reported in Figure 17a. It can be noticed that a slightly higher final positioning error is obtained, which is found equal to 13.76 mm. The average RMSE value is 5.27 mm.

This is very likely due to the floor typology that is more challenging as already stated. Nevertheless, the obtained errors are still in good agreement with the expected ones.

Finally, a continuous acquisition is performed for the experiment VI in order to evaluate the capability of method in estimating straight displacements. The cart has been moved at an average speed of 0.12 m/s by still following the linear stage. An overall distance of 3.18 m has been traveled. The result is reported in Figure 17b.

In this test, the final positioning error is equal to 40.64 mm. By comparing this error with the one of experiment V, one can notice that it is three times its counterpart. Although in this case the floor presents an higher number of visual features, even if of a small margin, the accumulated error increases, likely due to at least two reasons: 1) in experiment V images are acquired while the vehicle using the sensor is not moving while in VI it does; 2) a longer distance and more images are considered. Consequently, possible errors introduced during the motion estimation could involve drift of the trajectory from the expected one.

**Figure 18:**
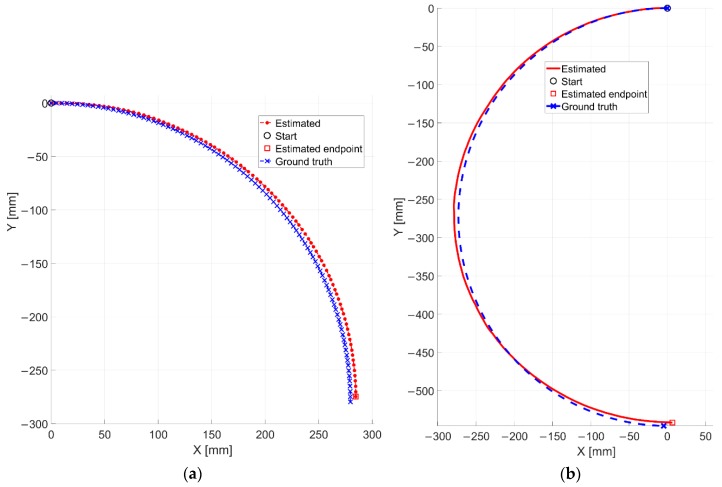
Estimated trajectories related to the (**a**) experiment VII and (**b**) the experiment VIII compared with the reference ones.

The proposed methodology has been evaluated by also considering pure rotational movements. The setup of Figure 9c,d is always considered. Furthermore, the very low textured industrial floor is taken into account. In the experiment VII, the camera fastened to the rotational stage by means of a metallic support, has been moved by performing a clockwise rotation of 90 degrees by a step of one degree. In Figure 18a is reported the result related to this experiment.

It can be observed that the estimated trajectory differs from the reference one by small margins. In this case, an average angular RMSE value of 0.39 degrees and a final positioning error of 4.01 mm are found, respectively. The traveled distance is of 0.44 m.

Finally, a continuous acquisition has been considered for the experiment VIII, where a 180 degrees rotation has been performed (see Figure 18b). The average angular speed is about 19.96 Deg/s. As for the previous test, the trajectories look qualitatively very comparable. For the sake of completeness, the final positioning error is also reported, which is found equal to 7.07 mm. The traveled distance is 0.87 m.

**Figure 19:**
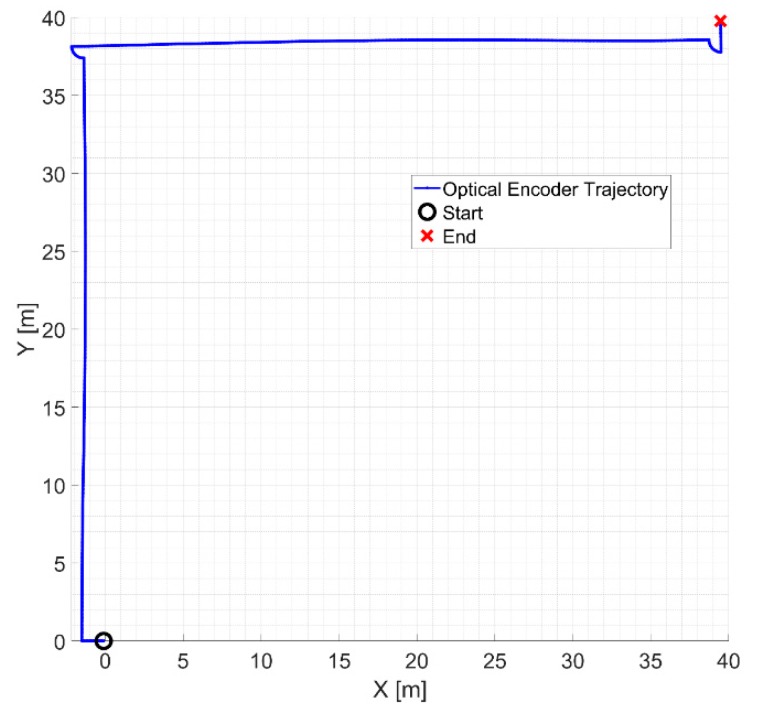
Estimated trajectory made by the AGV by using our vision sensor.

The last experiment has been performed by using the omnidirectional AGV for performing the movements. The AGV has traveled a distance of about 80 m at an average speed of about 0.6 m/s by performing straight displacements and regular rotations of about 90 degrees (see Figure 19). A final positioning error of about 60 cm is found.

Our vision system works at an average frequency of about 35 Hz when the full image resolution is considered. However, by opportunely selecting a region of interest (RoI), it is possible to increase the working frequency up to 70 Hz, thus enabling the AGV at traveling to the highest conceivable speed of 1.6 m/s.

### 4.3. Discussion of Results

Finally, a comparative analysis is also considered for proving the accuracy of our odometer. The outcomes related to all the previous experiments are summarized in Table 2. Different metrics are reported in order to compare our method with the others of the state of art. The best results are highlighted in bold. It should be noted that the experimental results reported in the table are referred to the authors data included in their work: in most cases, a direct comparison is indeed unfeasible since they use different sensors and/or setup configurations (viewpoint, illumination and so on). Results are reported here for aiding comparisons.

As one can notice, most of the values in the table are quite comparable. Our method mostly provides low final positioning error rates. Regarding to the second experiment, the percentage error is higher even though of a small margin. This is due to a higher cart speed that might have reduced the number of matched keypoints. It should be noted that our experiments are split according to the environment in which they have been undertaken. Particularly, in the experiments I and II, that have been performed in the robotics lab, we had the possibility to acquire the ground truth by using the Vicon system. In this context, it was possible to understand the behavior and system performance in following random and/or complex trajectories. In this situation, the system accuracy is comparable to the other works although the latter have been mostly done following smoother trajectories.

Regarding to the experiment III, it was performed in the corridor of our institute where the Vicon system was not available and we had the possibility to detect the ground truth in just few positions. The experiment was made with the three-wheeled cart, whose motion control is challenging. Nonetheless, while considering just the annotated positions, the relative positioning error was found to be lower with respect to the state of art.

Experiments in the industrial context proved much more challenging both for a lack of direct way to get the ground truth and the particularly uniform ground floor that is available there.

As mentioned, most of the other methods discussed employ auxiliary sensors and are not directly comparable with the results described in this work. An exception is related to [25]. In the latter, the low relative positioning error is due to the typology of adopted approach. Specifically, the authors employ an appearance-based approach for obtaining the data of interest. However, their approach is tested on high-textured surfaces. Moreover, to the best of our knowledge, the template matching requires more computational time than the features-based approaches, provides less accurate results and it is not rotation invariant [48,49,50], even though a direct comparison of the template matching using our acquired data has not been done yet.

Good performance of proposed method in terms of heading estimation is observable as well. Specifically, the heading RMSE value is far lower than the one of [13] whereas, the final heading errors of our method look very similar to the other works. To summarize, it can be stated that a final positioning percentage error of 0.21% on an average distance of 17.2 m is achieved. By considering a longer run on the industrial setting, a comparable result has been found (percentage error of 0.94% after about 80 m). Finally, the average relative positioning error is about 3%, which is still in good agreement with current state of the art.

The proposed solution shows promising results although is not exempt from some limitations. In fact, the algorithm might be in trouble in case non-textured or very highly reflective floors are handled. The SURF method could provide too few robust interest points or not enough reliable descriptors. Additionally, deformable surfaces might involve a performance degradation of our odometer in terms of accuracy which could result in higher drift positioning errors. In the near future, additional experiments will be held in order to better investigate these situations.

## 5. Conclusions

In this article, an accurate vision-based odometer has been presented for indoor mobile robot localization. A monocular camera looking toward to the floor is employed for acquiring the corresponding images. For each image, a feature-based approach enables to locate robust keypoints to be correlated with the ones of the consecutive images, captured during the AGV motion. By taking advantage of the point descriptors, statistical and geometric information, robust one-to-one matches between keypoints associated to consecutive frames can be identified. The relative affine transformation matrix, where the motion information is encoded, can be computed by means of the detected keypoint correspondences. Furthermore, the introduction of camera pose compensation allows to achieve better pose accuracies. The method runs on a dedicated hardware.

An extensive quantitative and qualitative validation of proposed methodology has been provided through controlled indoor tests. The vision system has been implemented onto an omnidirectional AGV, thus proving once more its effectiveness and accuracy in pose estimation when an actual application is considered.

Further activities will be led to the development of other methods and algorithms for managing texture-less surfaces. Some of the choices made in this paper such as the way horizontal and vertical components are treated separately in the histogram filter could be better investigated and compared with alternative solutions. At the same time, while in this work perspective issues are not considered explicitly, further work can investigate if better results can be obtained otherwise.

This work has been focused on the usage of monocular camera in order to add a vision component to an omnidirectional AGV that already has other sensors mounted on it as well. Therefore, future work will also be dedicated to the integration of the vision-based odometer with the rest of system.

Moreover, the powerful tools of deep learning (DL) will be also taken into account for the detection of keypoints or for the ego-motion estimation by directly processing pair of images given as input. Specifically, both supervised and non-supervised approaches will be explored for accomplishing these tasks.

## Figures and Tables

**Table 1:** Classification of the experiments according to the floor typology.

Section	Experiment	Images #	Cart	Floor	Illumination	Ground Truth	Reflectivity	Textureness
§ 4.2.1	I	8456	Three-wheeled	Wooden	Trasversal	Vicon	Low	High
	II	4538						
§ 4.2.2	III	3870	Three-wheeled	Raised	Trasversal	Ground markers	Average	Average
§ 4.2.3	IV	51	Four-wheeled	Industrial	Uniform	LRF, auxiliary stages	High	Low
	V	101						Very Low
	VI	1380						Low
	VII	91						Very Low
	VIII	450						Very Low
	IX	26776	OmniAGV			Ground markers		Low/Very Low

**Table 2:** Quantitative results of discussed experiments compared with the methods of the state of art. The best values for each estimate are in bold.

Method		Traveled Distance (m)	Positioning RMSE (mm)	Heading RMSE (Deg)	Final Positioning Error		Final Heading Error (Deg)	Relative Positioning Error (%)
					(mm)	(%)		
[13]		3.14	159.6	21.5	-	-	-	-
[12]		0.45	27.8	-	17.5	3.89	-	4.4
[25]		40	-	-	550	1.38	2.8	1.4
[46]		0.2	-	-	5.8	0.4	-	6
		0.3	-	-	8.6	0.18	-	5.7
[47]		6÷12	-	-	-	0.31÷2.12	-	-
[22]		10	-	-	270	2.7	1.1	4
		20	-	-	600	3	1.3	
		50	-	-	1650	3.3	2.2	
		100	-	-	3930	3.93	3.3	
Our	I	17.83	56.3	2.3	109.8	0.16	3.4	4.8
	II	19.27	56.5	1.4	114.7	0.3	0.9	9.6
	III	14.48	42.8	-	80.7	0.17	-	0.8
	IV	0.5	**0.49**	-	**0.35**	**0.07**	-	4.15
	V	1.24	5.27	-	13.76	0.22	-	1.69
	VI	3.18	-	-	40.64	1.18	-	1.18
	VII	0.44	2.84	**0.4**	4.01	0.12	0.72	3.9
	VIII	0.87	-	-	7.07	0.77	**0.67**	**0.77**
	IX	80.21	-	-	600.81	0.94	-	0.94
